# Treatment with Leflunomide in Conjunction with Glucocorticoids for Dogs with Immune-Mediated Polyarthritis Is Not Associated with Improved Outcomes: A Retrospective Cohort Study of 93 Dogs from Australia (2017–2024)

**DOI:** 10.3390/vetsci11110537

**Published:** 2024-11-01

**Authors:** Remon Wilson, Inar Swift, Mikaela Groth-Semple, Sabrina Lee, Tamara Dann, Ahmed Arafa, Curtis Poyton, Mary Thompson

**Affiliations:** 1 Queensland Veterinary Specialists, 53 Flinders Parade, North Lakes, QLD 4509, Australia; 2 Manly Road Veterinary Hospital, 219 Manly Road, Manly West, QLD 4179, Australia; 3 Department of Preventive Cardiology, National Cerebral and Cardiovascular Centre, Suita 564-8565, Japan; 4 Department of Public Health and Community Medicine, Faculty of Medicine, Beni-Suef University, Beni-Suef 62521, Egypt; 5 Poyton Group 6 Ettarre Street, Bracken Ridge, QLD 4017, Australia; 6 Sydney School of Veterinary Science, The University of Sydney, Camperdown, NSW 2050, Australia

**Keywords:** canine, immune-mediated, polyarthritis, leflunomide, prednisolone, synovial fluid, immunosuppressive, relapse, adjunctive therapy

## Abstract

Immune-mediated polyarthritis (IMPA) has a relatively high relapse rate compared to other immune-mediated diseases. Leflunomide is frequently used to treat dogs with IMPA in conjunction with prednisolone. This retrospective cohort study aimed to evaluate the therapeutic efficacy of leflunomide as an adjunctive therapy to prednisolone in reducing relapse and mortality rates in dogs diagnosed with IMPA in Australia. The medical records of client-owned dogs diagnosed with IMPA at a specialist referral hospital in Southeast Queensland from 2017 to 2024 were reviewed. A total of 93 dogs were included in this study, divided into two groups based on the treatment received: Group PRED, consisting of 53 dogs treated with prednisolone as the sole immunosuppressive agent, and Group L+PRED, consisting of 40 dogs that received leflunomide as adjunctive therapy alongside prednisolone. Data collected included breed, age, weight, sex, serum C-reactive protein concentration, results of synovial fluid analysis and microbial culture, treatment protocol, relapse rates and time to relapse, and mortality rates. There was no difference in relapse or mortality rates, time to relapse, nor time to discontinue prednisolone between the PRED and L+PRED groups. The L+PRED group had higher body weights and lower prednisolone dose rate at discharge compared to those in the PRED group. This study demonstrated that the use of leflunomide as an adjunctive therapy to prednisolone for the treatment of dogs with IMPA had no improved outcomes, reduced relapse rates, or shortening in the duration of prednisolone therapy when compared to dogs receiving prednisolone monotherapy.

## Simple Summary

Canine immune-mediated polyarthritis is a common disease that affects young dogs. This study aims at assessing whether leflunomide as add-on treatment to steroids would reduce the rate of relapse and improve survival in dogs with the disease. However, the results of this study suggest that there was no difference in outcomes between dogs that received or did not receive the drug as an add-on therapy. This could provide another step in understanding the nature of immune-mediated diseases and assist practicing veterinarians in making informed decisions about the most appropriate treatment for these conditions.

## 1. Introduction

Immune-mediated polyarthritis (IMPA) is a common immune-mediated condition of dogs [1]. Lameness, stiffness, reluctance to walk, joint effusion, and fever are the most common clinical signs [2,3]. Managing IMPA poses significant challenges due to the unpredictable nature of its clinical course [4]. One significant concern is the high relapse rate compared to other immune-mediated diseases [3]. Two recent UK studies reported relapse rates of 48% and 53% [5,6]. These frequent relapses often require prolonged immunosuppressive therapy, which can add to the complexity of treatment and represent substantial difficulties for veterinarians and owners. The same two studies reported high mortality rates of 17% and 19% [5,6]. This combination of high relapse rates and relatively high mortality highlights the need for improved therapeutic strategies in managing dogs with IMPA.

Corticosteroids, particularly prednisolone, are the mainstay of treatment for IMPA. The long-term use of prednisolone is associated with numerous side effects which negatively impact the quality of life for both dogs and their owners, prompting the search for prednisolone-sparing therapies [7,8].

Leflunomide, an immunosuppressive agent licensed in Australia and other countries for the treatment of human rheumatoid arthritis, has shown some potential in managing immune-mediated conditions including IMPA with an acceptable safety profile in dogs [9]. While some studies indicate the drug’s efficacy, their small sample sizes, design limitations, and inadequate focus on relapse rates undermine the overall strength of the evidence. A small case series of 14 dogs explored leflunomide as an alternative to corticosteroids, but it lacked follow-up data regarding clinical relapse after an initial improvement in only 8 dogs [10]. Additionally, leflunomide was included as part of the treatment in only some of the cases in one of these recent studies, and the study did not assess the impact of leflunomide on relapse or mortality rates [5].

Given these knowledge gaps, further investigation is necessary to assess the efficacy of leflunomide in improving outcomes in dogs with IMPA. Our study aimed to evaluate whether the use of leflunomide alongside prednisolone in dogs with IMPA was associated with a reduction in relapse and mortality rates compared with prednisolone monotherapy.

## 2. Materials and Methods

### 2.1. Study Design 

This retrospective cohort study evaluated the relapse and mortality rates in dogs with a clinical diagnosis of IMPA, treated with two different regimens: prednisolone as monotherapy (PRED) and a combination of leflunomide with prednisolone (L+PRED).

### 2.2. Data Collection 

Electronic medical records were searched for all dogs diagnosed with IMPA at Queensland Veterinary Specialists, a small animal referral hospital in Southeast Queensland, Australia, between January 2017 and April 2024. The keywords used were “immune-mediated polyarthritis”, “IMPA”, “polyarthritis”, and “polyarthropathy”. Records were reviewed and the following information was recorded for all dogs: breed, age, sex, weight, rectal temperature at presentation, hospital days and concurrent conditions. The treatment data collected were starting doses of prednisolone and leflunomide, duration of leflunomide and prednisolone treatment, total follow-up duration, occurrence of relapse, leflunomide and prednisolone dose at the time of relapse, and death due to the disease. When available, additional data collected included the results of synovial fluid microbial culture, serum C-reactive protein (CRP) concentration, and whether the IMPA was classified as associative or non-associative. 

### 2.3. Case Selection

The dogs were divided into two treatment groups based on the treatment received during the follow-up period of this study. Group PRED consisted of dogs treated with prednisolone as monotherapy and served as the control group for comparison with the alternative treatment group. Group L+PRED included dogs treated with leflunomide as adjunctive therapy to prednisolone. To be included in the L+PRED group, dogs must have received leflunomide in addition to prednisolone, either at discharge or during subsequent rechecks, but prior to any occurrence of relapse. Dogs were categorised as having associative IMPA if they presented with signs of resolvable comorbidities affecting areas distant from the joints, such as urinary tract infections (UTIs), acute diarrhoea, superficial pyoderma, or atopic dermatitis. Conversely, dogs were classified as having non-associative IMPA when no concurrent disease process was identified.

### 2.4. Eligibility Criteria 

All dogs enrolled in this study were required to exhibit one or more clinical signs consistent with IMPA, including fever (rectal temp ≥ 39.2 °C), shifting lameness, stilted gait, joint pain, and/or effusion. Dogs were required to have cytologic evidence of neutrophilic inflammation on synovial fluid samples from a minimum of two joints. Synovial fluid analysis was performed by a reference laboratory if there was sufficient sample collected in an EDTA tube. Alternatively, direct smears were made for cytological evaluation by board-certified clinical pathologists. Although detailed cytological evaluation data for each joint were not collected as part of this study, the cytological reports were reviewed by the authors and given scores based on the pathologist report interpretation (1 = mild; 2 = moderate; 3 = severe/extreme; or 4 = mixed but predominantly neutrophilic) and only dogs exhibiting predominantly neutrophilic inflammation of varying degrees in multiple joints were included. Dogs with only mononuclear inflammation were not included. When available, synovial fluid samples were collected for bacterial culture into blood culture bottles or transfer media swabs, either from a single joint, multiple joints, or pooled fluid from multiple joints.

Dogs were excluded from this study if they had insufficient medical records, no synovial fluid cytology was performed, or if they were lost to follow-up within 30 days. Dogs were excluded from both groups if they had received any immunosuppressive medication other than prednisolone in the PRED group or leflunomide and prednisolone in the L+PRED group prior to the end of the study follow-up period. Dogs were excluded from the L+PRED group if leflunomide was commenced following a relapse event. 

Dogs with other severe conditions potentially contributing to the development of IMPA were excluded, as these conditions could potentially affect the response to treatment. These conditions included concurrent neoplasia, systemic fungal infections, or dogs later diagnosed with septic arthritis with organisms not considered contaminants. However, dogs with concurrent clinical signs related to other non-fatal and non-severe concurrent conditions such as gastroenteritis with associated diarrhoea, pancreatitis, UTIs, oral ulceration, or dermatological conditions (limited to atopic dermatitis or superficial pyoderma) were not excluded but were categorised as associative IMPA. 

A dog was considered clinically relapsed if it showed one or more clinical signs of IMPA after previous resolution. Any escalation of immunosuppressive treatment was considered an indicator of a perceived relapse by the attending veterinarian. Treatment escalation was defined as restarting immunosuppressive therapy, increasing the dosage or frequency of the current medication, or switching to or adding an additional immunosuppressive medication. Confirmation of relapse was also obtained through abnormal synovial fluid cytology and/or elevated CRP levels whenever these diagnostics were employed. However, these diagnostics were not mandatory for defining clinical relapse.

### 2.5. Statistical Analyses

The differences in the mean values and proportions of characteristics between PRED and L+PRED groups were calculated using the *t*-test and chi-squared test, respectively. The Cox proportional hazards models were used to calculate the hazard ratios (HRs) and their 95% confidence intervals (CIs) of relapse and mortality. The follow-up period was calculated from the baseline date to the date of outcome or end of the follow-up, whenever came earlier. Statistical Package for Social Science (SPSS) 2013 (IBM SPSS Statistics for Windows, version 22.0, IBM Corporation, Armonk, NY, USA) was used for data analysis. 

## 3. Results

### 3.1. Study Population

Medical records of 142 dogs diagnosed with IMPA between January 2017 and April 2024 were identified. Records were reviewed for eligibility based on the diagnostic criteria for the disease.

From the PRED group, 34 dogs were excluded. Of these, 19 were excluded due to lack of follow-up, and 8 due to receiving a second immunosuppressive medication. The remaining 7 dogs were excluded despite initial diagnosis and treatment for IMPA, due to subsequent findings that compromised the accuracy of the diagnosis and treatment. These findings included positive synovial fluid cultures with organisms not considered contaminants (*Staphylococcus pseudintermedius* and *Pasteurella canis*) in two of the seven dogs, suspected synovial myxosarcoma in one, suspected endocarditis on echocardiography in one, disseminated fungal infection in one, and a history of total hip replacement in one. 

From the L+PRED group, 15 dogs were excluded. Nine dogs were excluded due to lack of follow-up, one for receiving additional immunosuppressive medication, and five had concurrent conditions that could affect the response to IMPA treatment. Among these five, one dog had a cardiac tumour with pericardial effusion, one had a total hip replacement, one died from presumed concurrent immune-mediated haemolytic anaemia (IMHA), one was later diagnosed with steroid-responsive meningitis arteritis (SRMA), and one died of unknown causes one week after diagnosis.

**Figure 1:**
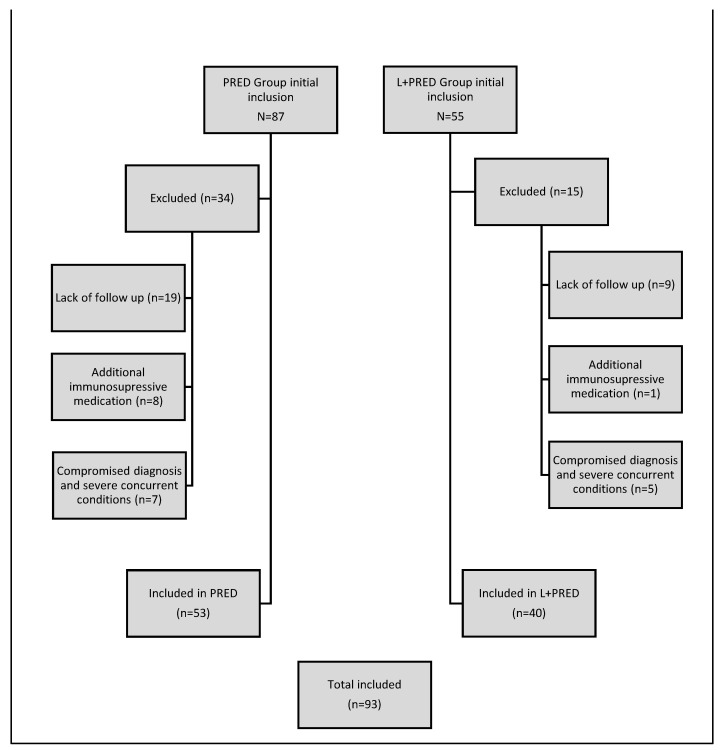
A flowchart summarising the exclusion process for each group.

A total of 93 dogs met the inclusion criteria for this study. These dogs were retrospectively assigned to either the L+PRED group (*n* = 40) or the PRED group (*n* = 53) based on the treatment protocol received (Figure 1).

### 3.2. Baseline Characteristics

Among the 93 dogs included in this study, there were 52 (56%) females (43 neutered) and 41 (44%) males (27 neutered). The most frequently presented breeds were Border Collie and English Staffordshire Bull Terrier (*n* = 8 each), Labrador Retriever (*n* = 7), English Cocker Spaniel (*n* = 6), Golden Retriever and German Shepherd dog (*n* = 5 each), Rottweiler and Kelpie (*n* = 4 each), and 27 breeds were represented with < 4 dogs. There were no differences in breed distribution between the PRED and L+PRED groups (*p*-value > 0.05). Breed distribution is provided in Supplementary Table S1. 

The sex distribution did not vary across groups: male neutered dogs comprised 26.4% in the PRED group and 32.5% in the L+PRED group, male entire 18.9% and 10%, female spayed 41.5% and 52.5%, and female entire 13.2% and 5%, respectively (*p*-value = 0.169). L+PRED dogs were 10 months older and 5 kg heavier than the PRED dogs (*p*-values = 0.183 and 0.033, respectively). A summary of age, sex, and body weight are presented in Table 1.

### 3.3. Body Temperature and Diagnostic Results

Body temperature (PRED 39.2 ± 0.7 and L+PRED 39.3 ± 0.8), number of joints sampled via arthrocentesis (PRED 2.9 ± 0.8 and L+PRED 3.1 ± 1.0) and the score of the synovial fluid cytology (PRED 1 = 0%, 2 = 30.2%, 3 = 66%, 4 = 3.8%; L+PRED 1 = 2.5%, 2 = 32%, 3 = 60%, 4 = 5%) did not vary between groups (*p*-value > 0.05). 

Synovial fluid microbial cultures were performed in 56/93 (60.2%) dogs (PRED 56.6% and L+PRED 65%). All microbial cultures, except for three in the PRED group and four in the L+PRED group, reported no bacterial growth. In the PRED group, the isolates identified were *Ralstonia insidiosa*, *Serratia marcescens*, and *Achromobacter* spp. In the L+PRED group, *Serratia marcescens* was isolated three times, and *Achromobacter* spp. once. All the positive bacterial cultures reported were regarded by the reference laboratory and treating veterinarian as most likely resulting from bacterial contamination during synovial fluid sample collection. 

CRP was measured on presentation in 18/53 (34%) dogs in the PRED group and 8/40 (20%) in the L+PRED group, with median values of 79.6 mg/L (IQR 51.3–95.5) and 78.2 mg/L (IQR 22.2–99.9), respectively. An abnormal CRP concentration was defined as greater than 10 mg/L.

### 3.4. Concurrent Conditions and Association to IMPA

The presence of concurrent dermatological conditions (PRED 17%, L+PRED 10%), diarrhoea (PRED 49%, L+PRED 30%), classification as associative IMPA (PRED 52.8%, L+PRED 32.5%), and duration of hospitalisation (PRED 2.2 ± 1.5 days, L+PRED 2.0 ± 1.3 days), did not differ between the groups (*p*-value > 0.05). Concurrent conditions other than diarrhoea and dermatological conditions are summarised in Table 2.

### 3.5. Treatment Data

Dogs in the PRED group were more likely to receive intravenous dexamethasone prior to oral prednisolone (PRED 75.5% and L+PRED 55.0%; *p*-value = 0.038) and received a higher initial dose rate (mg/kg/day) of prednisolone (PRED 1.7 ± 0.5 and L+PRED 1.5 ± 0.4; *p*-value = 0.010). However, there was no difference in the mean number of days on prednisolone (PRED 176.8 ± 198.9 and L+PRED 159.5 ± 109.9). In the L+PRED group, leflunomide was started at a mean dose of 1.9 ± 0.4 mg/kg/day within 9.9 ± 11.6 days from diagnosis. Immunosuppressive treatments and duration of prednisolone treatment are summarised in Table 3.

### 3.6. Relapse and Mortality Data 

The overall relapse and mortality rates for all 93 dogs in this study were 30.1% and 10.8%, respectively. There was no difference between the relapse rate (PRED 26.4% and L+PRED 35.0%) or the overall mortality rate (PRED 9.4% and L+PRED 12.5%) between the two treatment groups (*p*-value > 0.05). The median number of days to relapse was 141 (IQR 98–178) in the PRED group and 108 (IQR 54–139) in the L+PRED group, with no difference between the groups (*p*-value > 0.05). At the time of relapse, 16 (PRED 9 and L+PRED 7) of the total 28 relapsed dogs were receiving prednisolone at a mean dose of 0.5 ± 0.3 mg/kg/day. All of the 14 relapsed L+PRED dogs were receiving leflunomide at a mean dose of 1.7 ± 0.3 mg/kg/day at the time of relapse. Relapse was confirmed in four PRED and three L+PRED cases via repeat arthrocentesis, and elevated CRP confirmed the relapse in three PRED and one L+PRED cases. 

In the Cox regression analysis, the unadjusted, age- and sex-adjusted, and multivariable-adjusted models showed no additive benefit of leflunomide in reducing clinical relapse or mortality: HRs for relapse (95% CIs) were 0.88 (0.41–1.89), 0.76 (0.34–1.70), and 0.43 (0.16–1.14), respectively; for mortality: 1.02 (0.29–3.57), 0.66 (0.17–2.53), and 0.34 (0.06–1.86), respectively (Table 4). 

### 3.7. Adverse Effects Related to Leflunomide

Leflunomide-related adverse effects were suspected in 6 of 40 dogs (15.0%) in the L+PRED group. Diarrhoea occurred in 3 dogs; in 2 cases, it was self-limiting, while in the third, it led to discontinuation of the medication after 68 days. One dog experienced a transient decrease in appetite, which resolved without intervention. One dog developed a severe hepatopathy, with serum ALT concentration of 5122 IU/L (reference interval [RI]: 10–125) 57 days after starting leflunomide. This dog was euthanised due to suspected hepatic encephalopathy-induced seizures. Additionally, one dog developed pancytopenia with severe neutropenia (0.05 × 10^9^/L, RI: 2.95–11.64), thrombocytopenia (56 K/µL, RI: 148–484), and anaemia (haematocrit 30%, RI: 37–61) 17 days after initiating leflunomide. This dog recovered after discontinuation of the medication. Notably, none of the dogs that discontinued leflunomide were among the relapsed cases.

### 3.8. Subsequent Relapses 

While the Cox regression analysis included data only up to the first relapse, it is noteworthy that of the total 28 dogs that experienced a relapse, 12 (43%) experienced subsequent relapses ranging from two to five relapses per dog. Additionally, of the 14 relapsed dogs in the PRED group, 8 were later started on leflunomide at a mean dose of 2.2 ± 0.5 mg/kg/day. The median days to relapse and starting leflunomide of those eight dogs was 149 (IQR 112.5–237.5). Of these eight dogs, five experienced further relapses whilst receiving leflunomide. In the L+PRED group, 5 of the 14 relapsed dogs experienced at least one additional relapse while receiving leflunomide during the prednisolone tapering phase.

## 4. Discussion

We conducted a retrospective evaluation of the use of adjunctive leflunomide in the treatment of dogs with IMPA (L+PRED) and compared the outcomes of relapse and mortality rates to a control group that received prednisolone as monotherapy (PRED). Though focused on a single population, our study provides valuable insight into IMPA in Australia, where data on relapse rates have previously been lacking. While the L+PRED group showed slightly higher relapse and mortality rates, no statistically significant differences in these outcomes were observed. Therefore, the findings of this study do not support the routine addition of leflunomide to prednisolone in the treatment of IMPA.

Although this retrospective study has inherent limitations in fully understanding the factors influencing clinicians’ decisions to initiate leflunomide as a second-line treatment, it seems that body weight significantly influenced these decisions. The L+PRED dogs had a higher body weight and lower prednisolone starting dose rate. This likely reflects an attempt by clinicians to reduce prednisolone dosing in large breed dogs when adding leflunomide. Additionally, in our practice, clinicians tend to use a prednisolone dosing regimen of 40 mg/m^2^ per day in dogs over 15 kg instead of the commonly recommended immunosuppressive dose of 2 mg/kg/day in an attempt to minimise undesirable prednisolone side effects [9,11]. This could result in the overall lower prednisolone dose rate in the L+PRED group. Clinical impression of severe disease or slow resolution of clinical signs whilst in hospital is another factor that may have influenced the clinician’s decision to add leflunomide. 

The total relapse rate in our study was 30.1%, which was comparable to one recent and one older UK study [3,4]. The most recent study assessed the relapse rates in 32 dogs with IMPA compared to dogs with IMHA and immune-mediated thrombocytopenia (ITP). The study concluded that IMPA had the highest relapse rate, with 90% of relapses occurring within the first 12 months after diagnosis. This is comparable, albeit with a longer median time to relapse, to our study. Similar to our findings, the authors also found no difference in the use of combination immunosuppressive treatments between dogs that relapsed and those that did not across all disease groups, including IMPA [3]. 

Another recent UK study reported a relatively higher relapse rate of 48%. This study’s aims did not include assessing the impact of leflunomide on relapse rates; however, it did record that 14 out of 23 dogs received leflunomide alongside prednisolone, with 11 of the 14 experienced relapse [5]. A possible explanation for this study’s higher relapse rate could be the inclusion of dogs with systemic infections, other immune-mediated diseases, and neoplasia. Although it is difficult to compare outcomes between manuscripts, our study excluded any dog that could have any severe underlying disease. The number of presumed associative IMPA cases in our study appears relatively high; however, most of the associated conditions were non-severe and non-fatal and would not have been expected to impact on the treatment response. The nomenclature system of associative and non-associative was first proposed for IMHA in dogs [12], and it was later adapted for IMPA in a recent study [3], a classification preferred by the authors. However, the term can be broad, potentially leading the readers to assume that the associative IMPA cases in our study are directly related to other more severe diseases.

Assessing the efficacy of individual drugs or dose rates is challenging when medications are used in combination and usually requires prospective randomised controlled trials to evaluate each drug individually. Many prospective studies may have relatively short follow-up periods, limiting the ability to assess long-term relapse rates and outcomes with immunosuppressive drugs. While prospective studies may capture early outcomes, predicting relapse in dogs with IMPA remains difficult. This was shown in a well-designed 90-day prospective trial comparing prednisolone to cyclosporine in 20 dogs (10 per group) and found a 70% response rate to both treatments, highlighting early effectiveness but not long-term relapse risks [13]. The median time to relapse in our study was 141 days in the PRED group (IQR: 98–178) and 108 days in the L+PRED group (IQR: 54–139). This is longer than the 90-day follow-up duration of the aforementioned study, emphasising the need for long-term follow-up for studies to evaluate the relapse rate.

Similarly, the difference in disease duration may explain why leflunomide benefits humans with rheumatoid arthritis (RA) but not dogs with IMPA. The RA disease process in humans is chronic, requiring long-term management to decrease persistent symptoms, compared to canine IMPA, in which most cases achieve remission and do not require indefinite treatment. In dogs, the main concern is acute relapse during corticosteroid tapering, unlike the long-term disease control required in human RA. Many IMPA relapses are mild and can be managed by adjusting the prednisolone dose, while severe or recurrent cases may require addition of other immunomodulating medications [4].

The role of leflunomide in the treatment of more severe chronic and erosive forms of IMPA recognised as having a more similar pathogenesis to human RA has been minimally investigated in the veterinary literature in a small case series of 13 dogs with erosive IMPA, of which 9 were managed with a relatively high leflunomide dose (2.6 to 4.5 mg/kg/day) [14].

Leflunomide as an alternative to prednisolone has been also reported to provide disease control in 8 out of 14 dogs in a previous case series [10]. However, no long-term follow-up was performed to assess the relapse rate, and 6 out of the 14 (43%) did not respond adequately, representing a large portion of the population. Notably, this study was conducted in a different geographical region, and three dogs were seropositive for tick-borne infections and received doxycycline treatment, which may have influenced the outcomes. It remains unclear whether geographical variations in infectious disease prevalence, in particular tick-borne disease, play a role in the outcomes of IMPA treatment [1,15].

It should also be noted that the cases included in our study received a slightly lower initial leflunomide dosage (1.9 ± 0.4 mg/kg/day) than advocated in some previous studies [10,14,16]. The rationale for using a lower leflunomide dosage is not solely anecdotal. The authors observed fewer adverse effects without a difference in outcomes. These findings were also supported by a retrospective study recommending a starting dose of 2 mg/kg/day, which found significant median dose differences between dogs with adverse events (2.9 mg/kg/day) and dogs without adverse events (1.6 mg/kg/day). The same study also found no difference in the median dosages between dogs with immune-mediated diseases that responded (1.9 mg/kg/day) and those that did not respond (1.7 mg/kg/day) [17].

Several limitations arise from the retrospective nature of this study. Firstly, the use of regression analysis does not provide any statistical basis for assessing subsequent relapses. Also, dogs that experienced repeated relapses may have been genetically predisposed to immune-mediated diseases or autoimmunity, re-exposed to an unidentified environmental or infectious trigger that initiated the disease or had an undiagnosed underlying condition. This subset of dogs may have been at higher risk for relapse, independent of the treatment protocol used. Furthermore, variability in treatment protocols such as drug combinations and differences in doses and durations limits a full assessment of leflunomide’s role. For instance, the L+PRED group received a lower starting dose of prednisolone compared to the PRED group, likely due to the higher body weight of the dogs in this group. While this could be a potential confounding factor that might obscure the effect of leflunomide, it is unlikely to have had a significant influence, as relapses in both groups occurred much later, when prednisolone doses were either low or discontinued.

Another limitation of this study is our inability to objectively evaluate whether leflunomide provided any clinical benefit in reducing glucocorticoid adverse effects in the L+PRED group compared to the PRED group. The presence of adverse effects based on subjective reports from dog owners was noted in the clinical records of dogs in both groups. A prospective study would be necessary to accurately quantify and compare adverse effects between the two groups, given that both were receiving glucocorticoids. Clinicians commonly initiated leflunomide at diagnosis with the aim of reducing prednisolone-related side effects. However, our data indicated that leflunomide did not significantly reduce the duration of prednisolone treatment, which we believe would be necessary to meaningfully decrease prednisolone-related side effects.

Moreover, while radiography was not part of the eligibility criteria in our study, it may have offered further insights into the potential presence of joint erosion. Nonetheless, the lack of radiography in both groups is not expected to have influenced the results. Finally, in most cases, relapse assessment relied on the recurrence of clinical signs and the response to therapy adjustments, rather than definitive cytological confirmation. Although serum CRP concentration quantification was not available in our practice before 2020, it has now become readily accessible, offering a valuable non-invasive tool to help identify relapse in dogs with IMPA [18].

## 5. Conclusions

Our study findings indicate that leflunomide did not offer a therapeutic advantage to reduce relapse rate compared to prednisolone as monotherapy within this cohort. When considering the costs and potential adverse effects of leflunomide, one should question using the drug as an adjunctive therapy at the time of diagnosis for non-relapsed canine IMPA cases. A prospective randomised controlled trial involving a higher leflunomide dose may be necessary to thoroughly evaluate the long-term efficacy in dogs with IMPA.

## Figures and Tables

**Table 1:** Summary of age, body weight, and sex distribution.

Characteristics	PRED *n* = 53	L+PRED *n* = 40	*p* Value	All Dogs *n* = 93
Age (years) ^1^	4.0 ± 2.9	4.9 ± 3.3	>0.05	4.4 ± 3.1
Weight (kg) ^1^	19.9 ± 10.6	25.0 ± 12.1	<0.05	22.1 ± 11.5
Sex ^2^			>0.05	
Male	24 (45.3)	17 (42.5)		41 (44.0)
Female	29 (54.7)	23 (44.3)		52 (56.0)
Neutered ^2^				
Yes	36 (67.9)	34 (85.0)	>0.05	70 (75.2)
No	17 (32.1)	6 (15.0)		23 (24.7)

^1^ Age and weight values calculated as mean and ±standard deviation. ^2^ Sex and neuter status provided as number of dogs and percentage (%).

**Table 2:** Summary of the number of cases with concurrent conditions (other than diarrhoea and dermatological conditions) per treatment group.

Condition	PRED	L+PRED
Pneumonia	2	
Mild anaemia	1	
Pancreatitis	1	1
Urinary tract infection	1	1
Sialadenitis	1	
Hookworm infestation	1	
Oral ulceration	1	1

**Table 3:** Immunosuppressive medication dose rates (mean ± standard deviation) and number of prednisolone days for each treatment group.

	PRED (*n* = 53)	L+PRED (*n* = 40)	*p* Value
Dexamethasone *	0.28 ± 0.09	0.44 ± 0.09	0.038
Prednisolone *	1.7 ± 0.5	1.5 ± 0.4	0.010
Leflunomide *		1.9 ± 0.4	
Prednisolone Days ^†^	176.8 ± 198.9	159.5 ± 109.9	0.623

* mg/kg/day. ^†^ Number of days (mean ± standard deviation)

**Table 4:** Association between adding leflunomide to prednisolone and the risk of relapse and mortality.

	Model I	Model II	Model III
Relapse	0.88 (0.41–1.89)	0.76 (0.34–1.70)	0.43 (0.16–1.14)
Mortality	1.02 (0.29–3.57)	0.66 (0.17–2.53)	0.34 (0.06–1.86)

Model I: HR (95% CI) unadjusted. Model II: HR (95% CI) adjusted for age and sex. Model III: HR (95% CI) adjusted for age, sex, weight, receiving dexamethasone, and prednisolone dose at discharge.

## Data Availability

The data used to generate the results in this manuscript can be made available if requested from the corresponding author. The data are not publicly available due to professional secrecy.

## Supplementary Materials

The following supporting information can be downloaded at: https://www.mdpi.com/article/10.3390/vetsci11110537/s1, Table S1: Breed distribution between the treatment groups.
