# Supplementary material for: Treatment with Leflunomide in Conjunction with Glucocorticoids for Dogs with Immune-Mediated Polyarthritis Is Not Associated with Improved Outcomes: A Retrospective Cohort Study of 93 Dogs from Australia (2017–2024)
Source: Vet Sci. 2024 Nov 1;11(11):537. doi: 10.3390/vetsci11110537 (PMC11599048; doi:10.3390/vetsci11110537)
Supplement: Supplementary file 1 [file vetsci-11-00537-s001.zip › vetsci-3242738-supplementary.pdf]

Table S1: Breed distribution between the treatment groups.

Breed<br>% within Group	PRED	L+PRED	Total
Border Collie	5	3	8
	9.4%	7.5%	8.6%
Labrador	2	5	7
	3.8%	12.5%	7.5%
English Staffordshire	6	2	8
	11.3%	5.0%	8.6%
Golden Retriever	4	1	5
	7.5%	2.5%	5.4%
Rottweiler	1	3	4
	1.9%	7.5%	4.3%
Cavalier King Charles Spaniel	1	2	3
	1.9%	5.0%	3.2%
Kelpie	2	2	4
	3.8%	5.0%	4.3%
French Bulldog	1	1	2
	1.9%	2.5%	2.2%
Jack Russell Terrier	2	1	3
	3.8%	2.5%	3.2%
Pomeranian	1	0	1
	1.9%	0.0%	1.1%

German Shepherd	3	2	5
	5.7%	5.0%	5.4%
English Cocker Spaniel	4	2	6
	7.5%	5.0%	6.5%
Whippet	1	0	1
	1.9%	0.0%	1.1%
Mini Fox Terrier	3	0	3
	5.7%	0.0%	3.2%
American Staffordshire	1	0	1
	1.9%	0.0%	1.1%
Boxer	2	0	2
	3.8%	0.0%	2.2%
Dalmatian	1	0	1
	1.9%	0.0%	1.1%
Maltese	1	2	3
	1.9%	5.0%	3.2%
Greyhound	1	0	1
	1.9%	0.0%	1.1%
Irish Wolfhound	1	0	1
	1.9%	0.0%	1.1%
Chihuahua	1	0	1

	1.9%	0.0%	1.1%
Lagotto Romagnolo	1	0	1
	1.9%	0.0%	1.1%
Beagle	1	0	1
	1.9%	0.0%	1.1%
Dachshund	2	0	2
	3.8%	0.0%	2.2%
Australian Cattle Dog	1	1	2
	1.9%	2.5%	2.2%
Neapolitan Mastiff	1	1	2
	1.9%	2.5%	2.2%
Poodle	0	3	3
	0.0%	7.5%	3.2%
West Highland White Terrier	0	2	2
	0.0%	5.0%	2.2%
Maremma	0	2	2
	0.0%	5.0%	2.2%
Standard Poodle	0	1	1
	0.0%	2.5%	1.1%
Bichon	0	1	1
	0.0%	2.5%	1.1%

Rhodesian Ridgeback	0	1	1
	0.0%	2.5%	1.1%
Mixed breed	3	2	5
	5.7%	5%	5.4%
Total Count	53	40	93
% within Group	100.0%	100.0%	100.0%
